# Extracellular Matrix Dynamics as an Emerging yet Understudied Hallmark of Aging and Longevity

**DOI:** 10.14336/AD.2022.1116

**Published:** 2023-06-01

**Authors:** Cyril Statzer, Ji Young Cecilia Park, Collin Y Ewald

**Affiliations:** Laboratory of Extracellular Matrix Regeneration, Institute of Translational Medicine, Department of Health Sciences and Technology, ETH Zürich, Schwerzenbach CH-8603, Switzerland

**Keywords:** extracellular matrix, collagen, matrisome, matreotype, geroprotector

## Abstract

The biomechanical properties of extracellular matrices (ECM) and their consequences for cellular homeostasis have recently emerged as a driver of aging. Here we review the age-dependent deterioration of ECM in the context of our current understanding of the aging processes. We discuss the reciprocal interactions of longevity interventions with ECM remodeling. And the relevance of ECM dynamics captured by the matrisome and the matreotypes associated with health, disease, and longevity. Furthermore, we highlight that many established longevity compounds promote ECM homeostasis. A large body of evidence for the ECM to qualify as a hallmark of aging is emerging, and the data in invertebrates is promising. However, direct experimental proof that activating ECM homeostasis is sufficient to slow aging in mammals is lacking. We conclude that further research is required and anticipate that a conceptual framework for ECM biomechanics and homeostasis will provide new strategies to promote health during aging.

## 1. Introduction: Aging and longevity

### 1.1. The human cost of aging

Until the beginning of the 19th century, the average human life expectancy was less than 30 years. By contrast, in 2015, that number more than doubled to 72 years [1]. We live twice as long as our ancestors did only two centuries ago on all continents [1]. What caused this dramatic increase in the lifespan of humans in such a short period in evolutionary terms? A large part of the answer is the increased awareness of public health, such as hygiene, better nutrition through the industrialization of food production, and medical advances such as the development of antibiotics and vaccines.

Today, age-dependent diseases such as cancer, diabetes, cardiovascular diseases, and neurodegenerative illnesses have become the predominant causes of death. Understanding the aging process has become a social imperative since the primary risk factor for developing any of the aforementioned illnesses is age itself. Curing one of the age-dependent diseases in isolation suffers from diminishing returns since the prolonged survival and age increase predisposes the individual to other age-dependent illnesses [2]. Even removing a single age-dependent disease like cancer altogether was projected to only increase the population's lifespan by approximately 2.2 years [3] due to the subsequent occurrence and higher probability of other age-dependent diseases [2]. In the 2021 analysis, Scott et al. demonstrated that compressing morbidity is more valuable than merely prolonging lifespan [4]. Targeting aging offers a more substantial economic boon than eradicating individual diseases. The World Bank assessed that increasing life expectancy by a single year by slowing aging is worth US-$38 trillion, more than the gross national income of the United States in 2020 of US $ 21 trillion [4, 5]. By studying the underlying mechanisms of aging, we aim to improve the quality of life and reduce the economic burden of age-dependent diseases.

### 1.2. What is aging?

“The general struggle for existence of animate beings is therefore not a struggle for raw materials—these, for organisms, are air, water, and soil, all abundantly available—nor for energy which exists in plenty in anybody in the form of heat, but a struggle for negative entropy…”

Ludwig Boltzmann (1974) [6]

Aging could be viewed as a fight lost to entropy [7]. The second law of thermodynamics states that the entropy of an isolated system that is not in thermodynamic equilibrium increases over time [6]. Life might be a continuous struggle to preserve order and fight entropy. Cells are open systems that utilize energy from their environment to create and maintain their ordered state [8]. Thus, increased entropy could manifest itself in an aged cellular state and ultimately results in cell death [9]. In light of this, we can define aging as the progressive accumulation of disorder (damage) leading to the loss of integrity and function of the organism resulting in its decreased probability of survival [7, 10].

We have yet to come up with a consensus on the definition of aging as our understanding of aging is an evolving process. Currently, there are more than a hundred competing theories of aging, and the three most widespread biological theories of aging are intertwined. First, natural selection-survival of the fittest concerning successful offspring-applies until reproduction, but in post-reproduction loses its selective pressure [11]. Thus, natural selection does not apply after the individual has reproduced. Second is the theory of antagonistic pleiotropy, which rests on the assumption that pro-growth genes are maintained in the population because they increase the individual’s fitness during its youth but can be disadvantageous for the individual post-reproduction [11, 12]. Third, postulated by Kirkwood in 1977, the Disposable Soma Theory states that organisms are restricted to finite resources to dispense for cellular processes for optimized fitness under natural selection [13]. As August Weissman stated that the germline is immortal, and damage to the germline could have fatal consequences for the next generation; maintaining the germline would be essential [14]. Therefore, an organism would allocate resources to preferentially maintain and repair damage in the germline rather than the soma leading to the accumulation of damage driving aging [13].

Taken together, these three theories of aging suggest different strategies to slow or even prevent aging. For instance, as stated by the antagonistic pleiotropy theory, genes that are good for development and growth during youth, might turn bad for an organism post-reproduction and accelerate aging. Thus, rebalancing these antagonistic gene products could promote health during aging. Similarly, the Disposable Soma Theory postulates that the maintenance mechanisms exist but need to be activated for somatic cells.

#### 1.2.1. The nine hallmarks of aging

Since its inception in 2013 by López-Otín, the nine hallmarks of aging have provided a constructional framework for aging research. The hallmarks of aging comprise: genomic instability, telomere attrition, epigenetic alterations, loss of proteostasis, deregulated nutrient sensing, mitochondrial dysfunction, cellular senescence, stem cell exhaustion, and altered intercellular communications. These hallmarks conjunctively link aging to molecular damage, repair, and systemic response processes. In a complex mammalian system such as ours, each hallmark should satisfy the following conditions: i) being present during "normal" aging; ii) experimental aggravation expedites aging; iii) experimental intervention is sufficient to decelerate the rate of aging, measurable for example as an increase in lifespan [10]. We will hark back upon these three criteria as we further discuss how each criterion is supported with experimental evidence.

In the instance of the genome, the DNA experiences constant damage and accumulates somatic mutations over time [15]. And the epigenetic patterns consisting of DNA methylation, histone modification, and chromatin remodeling are essential in regulating expression throughout life [16].

On the individual cell level, the obstacle that hinders the ability to faithfully preserve and access genomic information—such as damages to the DNA repair mechanism, telomere attrition, and epigenetic markers can all lead to aging. Proteins perform the bulk of cellular functions. And the processes involved in protein synthesis, regulation, and degradation are directly bound to cellular aging [17]. Damages to any of these functions negatively impact the integrity of secondary processes because they rely on the quality of these biological macromolecules [10]. Examples of such higher-level processes are cellular nutrient sensing and mitochondrial health. Nutrient-sensing allows cells to adjust their metabolism according to the levels of circulating nutrients and hormones-and this is a process closely tied to aging [18]. Mitochondrial damage accumulation can also drastically accelerate cellular decay [19].

On the tissue level, the consequences of the accumulated molecular damage result in stem cell exhaustion, stem cell senescence, and elevated levels of inflammation [10]. These damages ultimately lead to the disintegration of the intercellular communication of the whole organism [10]. While entropy affects all molecules, the nine hallmarks of aging appear to impose on the rate-limiting steps more than other biological processes.

To compensate for these age-related deteriorations, organisms have developed several mechanisms to maintain homeostasis by preventing, containing, or repairing the damage before leading to accumulation. One example of a protein achieving homeostasis is a molecular chaperone system called Hsp70. Hsp70 prevents damage by co-translationally assisting the folding of nascent polypeptides in the correct configuration by communicating with other proteins [20]. If precise folding is impossible, Hsp70 contains and relays the damaged protein to the proteasome for degradation [21, 22]. Hsp70 also repairs damage by separating toxic protein aggregates and targeting the toxic aggregates for degradation [23]. The aging rate of several organisms suggests investment in chaperone production shows a strong inverse correlation to the damage accumulation rate, repair, and aging [24-27].

Cells invest a large number of resources to power the safeguarding mechanisms for their biological macromolecules. The Hsp70 system requires adenosine triphosphate (ATP) hydrolysis to perform its role in protein quality control. Amongst the most costly maintenance mechanisms are proteostasis [28], lipid quality control [29], organelle and mitochondria turnover through autophagy [30], and mitophagy [31], respectively. Since animals that are already experiencing aging have less energy available to maintain their cellular structures [32], they accumulate more damage with time, contributing further to cellular aging—likely speeding up this vicious cycle [33].

#### 1.2.2. Temporal scaling

Extensive studies comparing long-lived and short-lived *C. elegans* survival distributions noticed that the main difference is the stretching or shrinking of time, *i.e*., temporal scaling [34]. Interestingly, the same underlying phenomena of temporal scaling have been observed with bacterial aging [35], potentially indicating a shared underlying process inherent to aging itself.

Both short and long-lived species exhibit many of the corresponding age-dependent physiological changes. In mammals, temporally scaled age-associated phenotypes include declining muscle strength, structural integrity, and increasing frailty [36]. Longitudinal expression profiles comparing wild-type to longevity mutant *C. elegans* also indicated temporal scaling of transcriptional signatures [37].

Ongoing clinical trial designs depend upon our current understanding of temporal scaling to quantify aging. Given longevity interventions are conserved, the applicability of temporal scaling in the healthspan of mammalian longevity interventions may also be conserved. In the instance of the Metformin in Longevity Study (MILES; NCT02432287)[38], the primary outcome is measured using RNA sequencing of muscle and fat tissues to fathom rejuvenation to a younger expression profile [36].

### 1.3. Longevity

“It is indeed remarkable that after a seemingly miraculous feat of morphogenesis, a complex metazoan should be unable to perform the much simpler task of merely maintaining what is already formed.” - George C. Williams (1957) [11]

Aging-the decline in function-depends on the ability of the organism to prevent and/or cope with the accumulation of damage [7, 10]. While historically, aging was considered predetermined and immutable, that generally accepted idea was directly refuted when empirical data showed a doubling of *C. elegans* lifespan through the intervention of a single gene acting on endocrine signaling [39]. This plasticity of the aging process enables longevity and allows a small number of individuals to reach much higher ages than the general population [40]. Moreover, in the geriatric *C. elegans* population, conditional depletion of the DAF-2/insulin/IGF-1 transmembrane receptor when 75% of the aging population had died enabled the nematodes to live twice as long in maximum lifespan as the untreated population [40]. Suggesting that the capacity and plasticity to promote longevity are preserved even when damages have already accumulated during very old ages.

We also observe that human aging is malleable. We all now live twice as long as our recent ancestors did about 100 years ago [1]. The New England Centenarian Study (NECS) found that 41% of supercentenarians required minimal to no assistance in daily activities, and 83% of supercentenarians in Okinawa did not show major age-associated declines up to age 105 [41, 42]. They are the prime example of the relationship between cellular maintenance mechanisms, the ability to modulate the aging process, and delaying the onset of age-dependent diseases [18, 43]. Many longevity-inducing interventions have been shown to up-regulate protective mechanisms, which further increase cellular maintenance and the ability of the organism to respond to stress [44].

#### 1.3.1. Interventions promoting longevity

Longevity can be induced by activating the preexisting maintenance machinery. A cell or an organism invests its energy either in growth processes or maintenance and repair. Several interventions have demonstrated that switching metabolism from growth to maintenance reinstates homeostasis mechanisms and increases lifespan in a diverse range of model organisms.

##### 1.3.1.1. Insulin and insulin-like growth factor-1 (IGF-1) signaling

The seminal discovery in 1993 that a mutation in a single gene, the insulin/IGF-1 receptor *daf-2* doubled the lifespan of *C. elegans* without displaying strong side effects [38] galvanized the field of aging research and highlighted insulin/IGF-1 signaling as the key mediator of longevity.

In *C. elegans*, signal transduced from the plasma membrane receptor DAF-2/insulin/IGF-1 receptor via the Phosphatidylinositol 3-kinase AGE-1 when perturbed increased in lifespan by 40% [45]. The insulin signaling propagates from AGE-1/PI3K phosphorylating the AKT-1/2 and activating the PDK-1 kinase to retain the transcription factors DAF-16/FOXO and SKN-1/Nrf1,2,3 in the cytoplasm [46]. Upon reduction of DAF-2/Insulin/IGF-1 receptor signaling, DAF-16/FOXO and SKN-1/Nrf1,2,3 translocate to the nucleus and activate a number of protective genes [47-49]. Furthermore, mutations in the homologous insulin signaling cascade in *Drosophila melanogaster* (*D. melanogaster*) have also shown significant increases in lifespan [50].

Insulin hormone signaling in mammalian cells is mediated by multiple receptors by binding to three different ligands—insulin, IGF-1, and insulin-like growth factor-2 (IGF-2) [51]. Insulin/IGF-1 is closely intertwined with growth hormone (GH) signaling in mammals. The insulin/IGF-1 pathway and its function in lifespan extension are conserved from invertebrates to mammals and in regulating lifespan extension [52, 53]. However, insulin receptor regulation in mice is more complex. Multiple studies with knockout mice have assessed the tissue-specific effect of insulin receptor loss on animal physiology. One study found the adipose-tissue-specific receptor knockout to be the only tissue-specific insulin receptor knockout with overall beneficial effects and led to an 18% increase in mean lifespan [54]. By contrast, disruption of non-neuronal insulin receptors, *i.e.*, peripheral tissue-specific loss of insulin receptors, in another study, failed to increase lifespan [55]. Furthermore, insulin receptor knockout in the liver is accompanied by severe adverse effects like glucose intolerance and insulin resistance [56]. Insulin receptor loss in skeletal muscle does not affect glucose metabolism but leads to increased fat mass as well as higher blood triglyceride levels [57] that are partially mirrored by the neuron-specific knockout model, which also leads to elevated body fat levels among other phenotypes [58]. These differences highlight the additional complexity in mammalian systems by tissue-specific roles of insulin signaling networks.

Inhibiting IGF-1 receptor function by administering IGF-1 receptor antibody to 19 months old mice was sufficient to increase lifespan [59]. Again, tissue-specific disruption of the IGF-1 receptor is complex, but brain-specific heterozygous knockout of the IGF-1 receptor increased the lifespan of mice [60]. This lifespan extension might be mediated through neuroendocrine signaling to systemically slow the aging of the whole organism.

Another example of lifespan extension is Ames and Snell dwarf mice through altered insulin/GH signaling. In these animals, the anterior pituitary is not formed correctly, and the animals experience an altered endocrine state lacking prolactin, growth hormone (GH), and thyroid-stimulating hormone (TSH) that induces hereditary dwarfism. Both strains display extraordinary longevity, with mean survival increases for Ames male mice of 49% and females 68% over control [61] and for Snell males and females, 23% and 25%, respectively [62]. The change in hormonal signaling also extends to insulin, with Ames dwarf mice displaying greatly reduced circulating levels of IGF-1 in plasma [63]. The GHRKO mice that lack GH receptors also display increased longevity ranging depending on gender and genetic background of 19-40% [64]. Taken together, the somatotrophic axis of the insulin/IGF-1 receptor and GH receptor couple nutrient-sensing to anabolic signaling to influence the aging rate.

##### 1.3.1.2. Dietary restriction

Dietary restriction (DR) refers to mostly non-genetic interventions that, in some form, lower the energy available to an individual without causing malnutrition by either changing the ratio of diet composition, feeding intervals, overall caloric content, or other means.

Studies in model organisms have highlighted the near-universal positive health benefits of DR. Multiple DR interventions in *C. elegans* increase lifespan significantly (>25%), acting through different pathways depending on the type of restriction applied [65]. Similar positive effects were also observed in *D. melanogaster* [66]. In a paired feeding study, where dogs received 25% less food from 8 weeks onwards, the restricted Labrador Retrievers significantly outlived their non-restricted pair-mates and had lower insulin levels. The DR dogs also displayed a delayed incidence rate of chronic diseases [67].

Multiple large-scale non-human primate studies on rhesus monkeys confirmed the beneficial health [68-70] effects of DR through reduced caloric intake. While the reduction in all-cause mortality was not conclusive, it was observed in a subset of studies, suggesting dependence on the food restriction regimen-including diet composition, duration of food availability, husbandry, and social structure [68, 69].

DR mimetics are considered a promising alternative in translating the health-beneficial effects of DR observed in model organisms. One candidate is Metformin-a first-line treatment drug for type 2 diabetes [71]. Metformin has been shown to induce a DR-like state and extend lifespan in *C. elegans* [72], *D. melanogaster* [73], *M. musculus* [74], and diabetic humans [75]. The currently ongoing TAME (Targeting Aging by Metformin) pilot study is the first of its kind clinical trial to assess whether Metformin gives meaningful improvements against pathological effects of aging on healthy individuals ranging from 55 to 74 years of age [76, 77].

##### 1.3.1.3. Signaling through mTOR

The mechanistic target of rapamycin (mTOR) is an evolutionarily conserved kinase that acts at the nexus of the cell signals related to growth, survival, and proliferation. It senses amino acid levels, growth factors in higher eukaryotes, including insulin/IGF-1, and nutrient availability for energy, such as glucose. Its downstream effects include the regulation of protein synthesis, autophagy, and glycolysis [78].

mTOR occurs in combination with different binding proteins and gives rise to two functionally conserved complexes, TOR complex 1 (TORC1) and TOR complex 2 (TORC2), in eukaryotes. TORC1 and TORC2 have distinct functions in the cell and are targeted differently by rapamycin [79].

*C. elegans* treated with rapamycin displays a significantly extended lifespan, likely affected by both TORC1 and TORC2 and dependent on the activation of the downstream transcription factor skinhead-1 (SKN-1)/Nrf2 and Activating Transcription Factor 4 (ATF-4) [48, 80-82].

In mice, a single three-month treatment of rapamycin initiated at 20-21 months of age resulted in a 16% increased median lifespan from birth and a 60% increase in median life expectancy after treatment compared to the vehicle control [83].

Although rapamycin is an FDA-approved drug, the recommendation of a lifelong regimen is hindered by strong adverse side effects. Questions remain in finding the optimal dosage, duration, and mechanism of this drug to assist healthy aging efforts [83].

##### 1.3.1.4. Lifespan extension through hormesis

Environmental stresses and hardships often dramatically reduce the survival of an organism. To defend against these challenges, many organisms devised a collection of stress-response mechanisms. However, the relationship between stress, protective response activation, and survival is not linearly connected-rather, it often follows an inverted U-shape or J-shape dose-response curve [84]. As such, within a hormetic zone of moderate and often transient stress, stressors are overcompensated by the protective response leading to an overall physiological improvement.

Hormesis-mediated lifespan extension has been studied in multiple species, including *S. cerevisiae* [85], *C. elegans, D. melanogaster* [86], *M. musculus*, *H. sapiens* populations [87], and human cells in culture [88]. In *C. elegans*, thermal stress [89, 90], hyperbaric oxygen [91], and NaAsO2 (aq) (sodium arsenite) solution [92] have been successfully linked to an increase in population survival. Previously described interventions like DR [93], can also be interpreted as a hormetic treatment.

Not all environment stresses—such as *C. elegans* exposed to UV and gamma irradiation [91] —activate a hormetic response, or they have not yet been tested in the correct dosage. Nevertheless, the concept that the protective response can overcompensate the original insult and result in a net benefit is an alluring concept for future rejuvenation treatments.

##### 1.3.1.5. Additional longevity intervention in C. elegans

Due to the ease of experimental manipulation, many additional lifespan-extending interventions have been discovered for *C. elegans*. Lifespan-extending interventions include hormetic oxidative stress treatment with either arsenite, peroxide, or juglone, somatic germline ablation either genetically using the *glp-1* notch receptor mutant [94] or laser ablation [95], altered bacterial food sources [96], increased proteostasis [97], over-expression of the energy sensor 5’ adenosine monophosphate (AMP)-activated protein kinase (AMPK) [98], hypodermal overexpression of Alzheimer-related protein (APL-1) [90], activation or overexpression of NADPH-oxidase and ROS signaling [99], perturbation of nicotinamide adenine dinucleotide (NAD) metabolism through the over-expression of sirtuins or feeding of the downstream metabolites nicotinamide and 1-methylnicotinamide [100], and a variety of compounds ranging from monosaccharides [101] to organic extracts [102-104], metabolic intermediates [105] to drugs [106]. Furthermore, epigenetic marks such as defects in trimethylation of histone H3 at lysine 4 (H3K4) have been linked to extended lifespan [107]. The interventions listed above fall within these broadly defined categories of DR, mTOR, AMPK, reduced Insulin/IGF-1 receptor signaling, sirtuins, stem cell ablation, protein turnover, epigenetic modifications, and chemical compounds.

#### 1.3.2. Longevity in the context of natural selection

If aging can simply be delayed through increased maintenance, why are these maintenance processes not activated more often? We can only speculate, however, as with any other phenotype, that natural selection favors the optimal tradeoff to maximize the fitness of each species. Increased energy expenditure on maintenance would logically require decreased investment in other advantageous processes like growth, reproduction, and others.

Maintenance, and thus the rate of aging, is one of many phenotypes that govern survival and reproduction. Thus, improving cellular repair is always a tradeoff between regulating aging and other investments.

Nutrient sensing, as one hallmark of aging, directly affects this resource allocation and displays one of the strongest effects on the lifespan of all age-regulating processes [10]. We might deduce that this is a self-preservation mechanism until the environment is again more hospitable. From this perspective, the ability of a single gene to be sufficient in doubling the lifespan becomes more plausible since it only requires changes in the regulation of already established repair processes.

In the wild type, the same repair processes are present but repressed because increased repair would put the organism at an evolutionary disadvantage (*i.e.*, Disposable Soma Theory). This greatly increases the prospects of longevity research since maintenance mechanisms do not need to be invented but need to be correctly activated [108].

## 2. Extracellular matrix dynamics during aging and longevity

Above, we introduced the theories of aging and the underlying molecular mechanisms that promote health during aging. Many of these molecular processes have been studied extensively for intracellular macromolecules and inter-tissue signaling. Although it is reckoned that damage accumulates in proteins outside of cells [109]-such as the extracellular matrix-less is known about how these extracellular proteins are maintained or repaired during aging. Below, we review the current literature and discuss emerging concepts of extracellular matrix homeostasis during aging.

### 2.1. Biological role of the extracellular matrix

The extracellular matrix (ECM) provides structural stability, anchors muscle fibers, forms a protective layer, shields cells from the environment, stabilizes and compartmentalizes organs, and mediates communication across the entire organism. The ECM is a highly dynamic structure. Its composition changes depending on the tissue, developmental stages, or other processes [110]. Together with its structural role, the ECM also determines the identity of each tissue [111]. The two main ECM categories are the basement membrane matrix and the connective tissue matrix—the basement membrane separates the stroma and epithelium, and the connective tissue matrix acts as structural support for the tissue.

The ECM directly controls the potency and differentiation characteristics of the cells in its vicinity. Cells change their behavior if removed from or placed into a different ECM. Cancer cells lose their carcinogenicity when transferred to an embryonic ECM [112]. Young ECMs can revert the senescence phenotype of cells [113] and rejuvenate older stem cells [114]. Conversely, young muscle tissue ages prematurely when implanted into the ECM of an older rat [115].

The differences in the ECM structure, composition, elasticity, and other cues were further investigated through the analysis of the behavior of tumor cells and stem cells in their respective ECM. Experiments involving engraftment of human colon carcinoma cells on BALB/c nude mice suggested that the ECM present in the implant location affected the ability of the cancer cells to metastasize—demonstrating that the local factors in the organs affected the production and secretion of tumor ECM-degrading enzymes [116].

The dissimilarities in colonization by circulating tumor cells showed the organ-specificity of ECMs. When comparing the soluble fraction of murine kidney and lung ECMs, the lung-colonizing cancer cell line displayed organ-specific preferential motility towards the lung extract over other tissues—suggesting chemotactic factors may be at play [117]. These experiments highlight the tissue-specificity of the ECM, its crucial role in cell-matrix signaling, and tissue aging.

### 2.2. Age-dependent deterioration of the ECM

The aging ECM is inundated by compounding maintenance issues. One hallmark of the aging process is the progressive stiffening of most tissues rich in ECM. Structural and functional changes of the matrix components, such as collagens, laminins, and elastins that come with an age-related loss of the enzymatic digestibility of the ECM show strong correlations to processes involved in the occurrence of many diseases [118-120]. These proteins are responsible for the elasticity, strengthening, and diffusion of nutrients and metabolites between the blood and the tissue cells [121]. Collagen networks work as tension elements, and the interfibrillar matrix is the compression element. The collagen fibrils serve mechanical properties that evolve with the maturation of the tissues in the presence of enzyme-induced covalent cross-links between molecules [62, 122, 123].

As long-lived proteins, collagens, and elastins often experience age-dependent modifications like the advanced glycation end-products (AGEs) [124]. This process involves slow non-enzymatic reactions between amino acids and sugars. And these reactions lead to AGEs like the pentosidine moieties [125]. The glycation products result in matrix stiffening, thus preventing enzymatic cleavages necessary for remodeling. This issue of AGEs formation with increasing age is exacerbated by diabetes, where the level of protein glycation is elevated and results in numerous complications-including reduced wound healing [126]. The AGE crosslinks likely alter the mechanical properties of the ECM proteins, such as collagen, making stiffer tissues with decreased viscoelasticity. This process can be involved in various diseases, like atherosclerosis and neuropathy [127].

Independently, mechanical and biochemical damages occur in the ECM, causing progressive collagen fragmentation during aging. These fragments and many proteins released into the extracellular space display poor solubility and often form aggregates. With these changes, a vicious cycle initializes when cells detect the changes in the matrix through integrin attachments and respond by secreting more modified enzymes like matrix metalloproteinases (MMPs) and other matrisome components [128]. In contrast to young fibroblasts, aged human skin fibroblasts express more matrix metalloproteinases-1 (MMP-1). Treating a young ECM with MMP1 results in fragmented collagen matrices resembling an old ECM. Furthermore, when fibroblasts are transferred to an ECM fragmented by MMP1 treatment, fibroblasts respond with elevated levels of MMP-1 expression, perpetuating the cycle that leads to further matrix fragmentation [128].

In the case of AGEs, neighboring cells respond with elevated protease levels to reduce matrix stiffness. However, the AGEs prevent proteolytic cleavage that may result in damaged non-glycated structures [129]. Thus, in an attempt to rebuild the ECM, the matrix is further damaged by uncontrolled protease activity and undirected collagen deposition [128]. These accumulated damages trigger an inflammatory response that can lead to fibrosis [130]. The fibrotic tissues that experienced extensive undirected ECM degradation and deposition leave the tissues scarred with decreased function. The biomechanical properties are transformed-with tissues being stiffer, less elastic, less organized, and weakened. The matrix progressively loses its ability to maintain its integrity and its microenvironment.

With the progression of age, this process can be observed in multiple human organs. In the heart, collagen deposition and fibrosis are critical determinants of cardiac output, and these elevate the risk of stroke due to higher myocardial stiffness [131]. In human skeletal muscle, increased collagen deposition results in higher tissue stiffness [132]. And such vascular alterations result in damage to multiple organs ranging from kidneys [133] to the brain [134]. Pulmonary fibrosis can occur when collagen synthesis and degradation lose their homeostatic balance causing devastating effects on the lungs. In the largest organ of the human body-the skin-opposing effects are observed. Observable wrinkles are the consequence of continuously decreased collagen content [135]. In aged human skin, collagen production and the fraction of the cell surface attached to the collagen fibers are reduced [136]. Thus, with tissues following different trajectories, generalization cannot be stated on the role of the ECM regarding tissue aging. Because the aging ECM is intimately linked to inflammation, aging itself then becomes the systemic determinant of tissue function-making rate regulation of matrisome synthesis and degradation vital to healthy aging.

Taken together, ECM accumulates damage during the aging process, decreasing its functionality and thereby fulfilling the first criterion of the hallmarks of aging.

### 2.3. ECM dynamics in disease progression

Deviations from ECM homeostasis may result in numerous pathologies. However, by investigating the mechanisms behind its foundering, many of these pathologies may be avoided. Herein, we explore various ECM-related pathologies and notably understudied gender differences in pathology progression, which demand further attention. In particular, exhibitions of notable organizational and compositional changes in post-menopausal women with increments in collagen, laminins glycosaminoglycans, and the waning of fibronectin [137]. This, concomitant with changes in the ECM and protease-related gene expression implying transcription machinery directly affected by aging [137] has largely been ignored. In another instance, however, Tyshkovskiy et al., have demonstrated the feminizing effect of caloric restrictions, growth hormone regulation, and other longevity interventions/drugs on male mice gene expressions that have been shown to diminutize the sex-specific differences in longevity interventions [138]. These seemingly conflicting results highlight the increasing need for more studies conducted on both sexes to better capture the entirety of ECM-related pathogenesis.

#### 2.3.1. Fibrosis

Loss of ECM homeostasis and the resulting excessive amorph deposition of collagen is observed in many fibrotic disorders [139]. In this process, unrectifiable structural damages to ECMs are inflicted, and also wound healing properties are distorted, which reduces tissue function [140, 141]. Moreover, inflammation proceeds fibrosis [142], and inflammation increases during aging (inflammaging) [143]. Even though fibrosis underlies approximately 45% of all mortalities in the U.S. alone [144], we have yet to develop a reliable early diagnostic biomarker. Development of novel methods is required to reverse fibrotic scarring and restore organ function that is the cause of such detriment [141, 145].

##### 2.3.1.1. Liver failure

Liver fibrosis is caused by chronic liver damage and protein accumulation in the ECM [146]. Despite the liver’s unique regenerative properties, persistent assault on the liver impels fibrosis and causes scar formation. ECM deposition-ultimately causes hepatic stellate cell failure [147]. Most fibrosis therapeutic strategies involve suppressing excessive collagen production, targeting ECM degradation, anti-inflammatory liver protection, and developing hepatic stellate cell inhibitors. Gene therapies are also being developed to directly target fibrosis [148].

##### 2.3.1.2. Heart failure

The ECM provides structural integrity, transmits signal transduction to cardiomyocytes and vascular and interstitial cells, and prompts force transmission. Disturbances in the cardiac ECM may cause myocardial matrix stiffening and thereby preserved ejection fraction heart arrest, reduced ejection fraction heart arrest, and electric conductance disturbance-all of which can lead to mortality [149, 150]. Changes related to age in cardiac ECM differ between sexes, with aged male rats exhibiting more fibrotic hearts than their female counterparts [151, 152]. Despite both sexes showing interstitial fibrosis, different sexes showed distinct Transforming growth factor beta (TGF-β) signaling pathways [153]. Implications of these gender-specific differences warrant further investigations.

##### 2.3.1.3. Lung failure

Tenascin-C and versican-a chondroitin sulfate proteoglycan-have been shown to be highly expressed in defective lungs. These defective lungs were ineffective at wound healing and were shown to lead to fibrosis [154] and provide evidence of the association of loss of ECM homeostasis with the pathology of lung fibrosis [155, 156].

##### 2.3.1.4. Kidney failure

Kidney ECM is composed of elastin, glycoprotein, proteoglycan, and collagen network-forging interstitial and basal matrices [157]. Chronic kidney disease patients have been observed to have calcification of ECM in soft tissues [158]. Nearly all chronic kidney disease patients progress to the stage of kidney fibrosis, and it is estimated to affect approximately 12% of the world with an equally high mortality rate [159, 160].

#### 2.3.2. Mechanotransduction failures

The mechanical properties of the ECM and its chemical and structural composition are dictated by cell-matrix interaction. Cells require sensing and regulation of ECM mechanics to preserve tissue-level functionality and structural integrity. Further studies are required to understand better how cell-matrix interactions affect mechanical homeostasis [161].

##### 2.3.2.1. Cancer

Cancer, characterized by uncontrolled proliferation and abnormal cell growth, in 2020 alone affected approximately 18.1 million people globally [162]. The ECM is a crucial requirement in the formation of the cancer microenvironment [163]. Increased ECM depositions and crosslinks have been attributed to tumorigenesis and its metastasis [164] by secreting the chemical and physical signals required for cancer development and invasion [165]. Interstitial matrices are predominantly formed by stroma [166]. Interestingly, for cancer progression, ECM remodeling is essential. Naba et al. confirmed through the study of the matrisome that cancer stroma includes fibroblasts [167]. These fibroblasts, in turn, regulate the deposition and remodeling process of the ECM [168].

Furthermore, tumor cells have been found to impose a pivoting influence on the deposition of fibronectin, collagen, and tenascin C [169, 170]. A compounding amount of evidence now suggests ECM deposition stimulates tumorigenesis and metastasis by assisting cancer cells to replicate and survive [171]. However, more studies are needed to understand the exact mechanisms of ECM produced by tumor cells and its role in the remodeling of the microenvironment to enable a targeted approach to cancer treatment [172].

##### 2.3.2.2. Loss of cellular identity

During aging, the dermis undergoes remodeling of the ECM and impaired cellularity [173, 174]. However, little is known whether this is attributed to the changes in the fibroblasts. Salzer et al. investigated whether different diets contributed to altered dermal fibroblasts in murine and its impact on lifespan. It was demonstrated that as time progressed, the definition of old fibroblasts became occluded, and even when present on the youthful dermis, fibroblasts were still indistinguishable. Old fibroblasts also showed decreased gene expression of ECM formation. But quizzically, these old fibroblasts contradictorily gained adipogenic traits similar to that in the neonatal state [175]. However, when placed on caloric restriction, these adverse effects were either reversed or mitigated. But in the instance of the high-fat diet, it accentuated the negative effects of aging. These results point to the loss of cell identity as one of the underlying mechanisms of aging [175-177].

##### 2.3.2.3. Stem cell exhaustion

Stem cell exhaustion is one of the aging characteristics and is a major culprit in age-related tissue regeneration reduction [178, 179]. The induction of *in-vivo* stem cells has supported the above assertion by restoring phenotypes associated with age-related decline in murine [180-182]. And it has been demonstrated that, while only existing in small numbers across various tissues and organs, tissue stem cells have an indispensable role in maintaining homeostasis and tissue regeneration [179, 182, 183]. Importantly, the ECM influences the regulation of stem cell pluripotency, stem cell differentiation, tissue repair, and regeneration [184]. Tenascin-C, in particular, is expressed primarily by bone marrow niche cells, which facilitate the regeneration of hematopoietic stem cells [185]. And these findings suggest stem cell rejuvenation can be potentiated for novel therapy by manipulating the mechanisms of ECM [183, 186].

##### 2.3.2.4. Cell death

Cell death is critical to body development and maintaining homeostasis to avert disease occurrence [187]. Cell death can be classified as non-programmed or programmed depending upon associated regulatory processes [187, 188]. Non-programmed cell death (Non-PCD)-necrosis-is irreversible cell damage induced by the extreme physical or chemical environment [189]. Programmed cell death (PCD) can be further subclassified into lytic and non-lytic cell death [190]. Lytic cell death comprises necroptosis and pyroptosis [191]. Non-lytic cell death, such as apoptosis, is critical in the maintenance of tissue organization and sustaining adequate cell count [192]. Rearrangement of tissue organization requires ECM, and the ability of the ECM to remodel is key to cell regulation [193]. Published findings have reported apoptosis as an effectuate of several diseases, including those in the kidneys [194, 195]. Apoptosis does not induce an inflammatory response, and phagocytes are responsible for clearing the produced apoptotic bodies [187, 196]. In a rat kidney study, glomerular cell apoptosis has been attributed to glomerular cell deletions and ECM accumulation, causing glomerulosclerosis [197].

Mesangial cells were suppressed from apoptosis by the basement membrane matrix and induced survival. However, apoptosis was increased with the suppression of antisense oligonucleotide signals opposing β1 integrin. These findings suggest mesangial cell regulation is dependent on the encompassing ECM via integrins [192]. Because mesangial cell propagation is closely related to apoptosis, a novel therapeutic strategy employing apoptosis manipulation for renal diseases can be expected in the future [192].

##### 2.3.2.5. Loss of mitochondrial homeostasis

Mitochondria are multifaceted essential organelles responsible for various tasks such as apoptosis, cell metabolism, proliferation, intracellular calcium homeostasis modulation, and ATP production [198-200]. Because of its many duties, any dysfunction in the mitochondria can have a devastating effect on human health. Numerous recent studies have pointed to the loss of mitochondrial homeostasis as the culprit in cancer development and progression [201, 202]. The ECM is also abundant in the cancer microenvironment, affecting its initiation and progression and leading to metastasis formation [169, 198].

There exists a bidirectional relationship between the ECM and mitochondria. Cell-ECM interaction modulates mitochondria directly or via actin cytoskeletons [203]. This interaction is particularly pertinent in the metastatic event where mitochondria enable cell migration as the primary energy source [198]. Meanwhile, mitochondria depolarization and oxidative phosphorylation influence the ECM through cell-ECM adhesion and MMP-dependent ECM degradation [202]. Moreover, mitochondrial homeostasis and stress responses (mitohormesis) are interlinked with ECM-integrin signaling in *C. elegans*, and human stem cells, and at least in *C. elegans* play an important role in healthy lifespan extension [204-207]. Further investigation is needed to understand the mechanism behind ECM involvement in mitochondrial energy production. In the future, possible therapeutic strategies may be employed which exploit the relationship between the ECM and mitochondria for cancer cure [198].

##### 2.3.2.6. Osteoarthritis

Osteoarthritis is the most common joint degenerative disease affecting about half of the population over the age of 65 [208, 209]. Abnormal bone remodeling and articular cartilage degeneration are often attributed to osteoarthritis, causing pain, and severe life impediments physically and financially in the aging population [210-213]. While aging has been attributed to the etiology of osteoarthritis, the exact mechanism of its pathogenesis is still not well understood [214]. Aging deregulates the homeostasis of the ECM and causes loss of integrity and deterioration of its architecture. These properties are seen as the causation of osteoarthritis with its increased inflammatory expression, loss of regenerative ability in tissues, and cellular senescence. In particular, chondrocytes are quiescent cells entrusted with cartilage formation that are thought to initiate and affect the progression of osteoarthritis [209]. It has been observed that during the disease progression, chondrocytes promote ECM malformation, forming clusters [215]. As age progress, chondrocytes affect proteoglycan synthesis and disrupt their composition [216]. Women have a higher incidence of osteoarthritis than men and are more heavily affected by the disease. It has been proposed that sex and genotypes of the chondrocytes affect the synthesis and catabolism of ECM microarchitecture, which creates differences in how the disease manifests depending on gender [217].

##### 2.3.2.7. Progeria

There is a potential link between premature aging disease and ECM. Agglomerative hierarchical clustering of the aging phenome and progeric diseases included Marfan and Ehlers-Danlos syndromes, two classical genetic ECM diseases besides three mitochondrial diseases [218]. Furthermore, Hutchinson-Gilford Progeria (HGPS) patient fibroblasts show dysregulated gene expression of ECM genes [219]. In premature aging HGPS disease mice models and also in human HGPS progeric cells, Wnt signaling is defective, resulting in a failure to produce a proper ECM by HGPS cells [220]. Strikingly, in genetic mosaic progeric mice models, wild-type cells secrete ECM proteins that build a normal ECM surrounding also the defective progeric cells [221]. In these mosaic mice, the proper ECM rescues the accelerated senescence and progeria, resulting in a normal lifespan [221]. This suggests that a faulty ECM is an important driver of progeria and accelerates aging. Furthermore, mice lacking membrane type-1 matrix metalloproteinase-14 (MT1-MMP4) that normally cleaves extracellular collagen matrices show accelerated senescence and short lifespans [222]. Similarly, mutating collagen type 1a1, so that MMPs fail to cleave and degrade collagen type 1a1 from the extracellular matrix, results in accelerated aging, age-related pathologies, and shorter lifespan [223]. This suggests that impairing ECM remodeling and homeostasis aggravates aging pathologies.

Taken together, a dysfunctional ECM can drive and accelerate age-related pathologies and, thereby, the aging process-fulfilling the second criterion of the hallmarks of aging.

**Figure 1.**
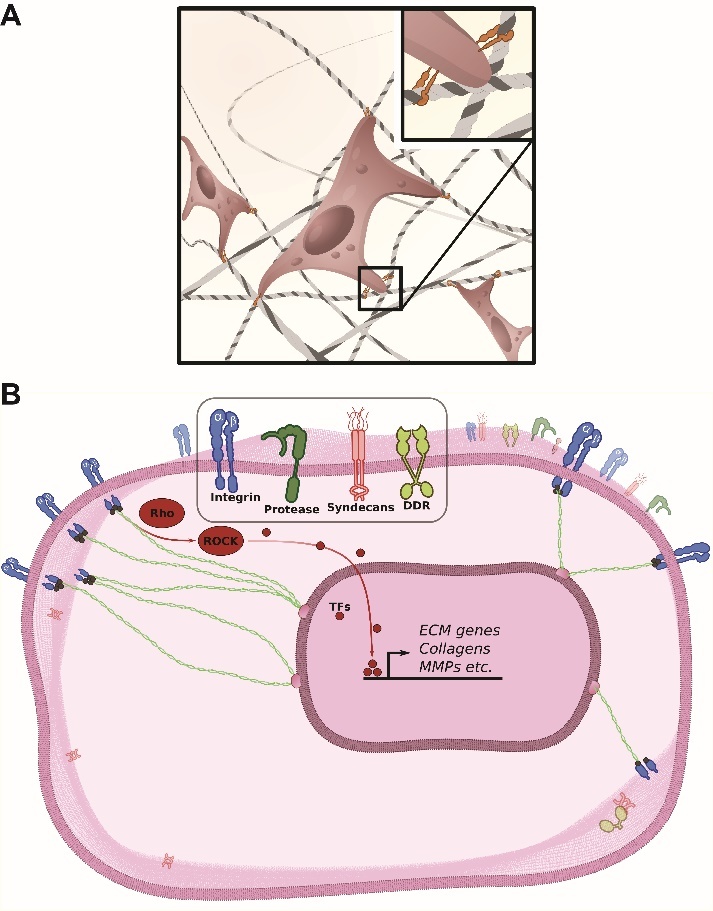
**ECM-cell and cell-ECM communication.** (A) Schematic illustration of fibroblasts attached to the ECM through integrin receptors. A magnified inset displays the heterodimeric cell surface receptors attaching to collagens in a highly simplified manner. (B) The inside-out and outside-in signaling relays through the plasma membrane (PM) of a typical cell. Four families of transmembrane proteins which are frequently involved in mechanotransduction are displayed (integrin, protease, syndecan, DDR)

### 2.4. Cell - ECM & ECM - Cell Communications

Cells do not exist in isolation (Fig. 1A). From *C. elegans* to humans, cells constantly exchange signals with their engulfing matrices and neighboring cells. Chemical cues like matrix-resident proteins or hormones offer one avenue of communication. The ECM strictly controls the storage, release, and passage [224].

Another way of relaying information is through direct contact mediated by a diverse array of adhesion proteins on the cell surface. A large part of cell-cell and cell-matrix communication in mammals is established through integrins, cadherins, selectins, and the immunoglobulin superfamily (Ig-SF). Adhesion molecules-which are all transmembrane proteins-constitute the family of cell adhesion molecules (CAMs) [225].

Binding and information exchange between the cell and the ECM is maintained by the integrin and discoidin domain receptors (DDRs). Integrins bind to collagens, fibronectin, elastins, and laminins. The discoidin domain receptors (DDRs) attach to fibrillar collagen—specifically collagen triple helix motifs [226, 227] (Fig. 1A). Integrins and DDRs mediate cell-ECM and ECM-cell signaling and are of crucial importance for cellular processes like survival, migration, differentiation, and the metabolic state of each cell. By extension, integrins play a vital role in the development, function, and integrity of all tissues. Integrins and DDRs are the predominant forms of collagen cell surface receptors in vertebrates, and they each bind to distinct collagens-raising a specific response [228].

Fibroblasts navigate and sense the ECM through multiple transmembrane receptors. These receptors link the structural matrisome components-such as collagen-and bundle to the cytoskeleton and vice versa, couple cytosolic signaling mechanisms to the extracellular space through cytoplasmic integrin regulation. In this way, the biomechanical properties of the ECM can be converted to signaling cues within each cell and translated into specific cellular behavior and maintain the identity of each cell attached to this particular ECM region. The information is relayed from the plasma membrane (PM) to the nucleus through small Rho GTPases and transcription factors-leading to changes in gene expression (Fig. 1B).

#### 2.4.1. Discoidin domain receptor family

The discoidin domain receptor (DDR) family consists of two members-DDR1 and DDR2. The DDR1 and the DDR2 are dimeric receptor tyrosine kinases (Fig. 1B) that modulate cell migration, adhesion, invasion, proliferation, morphogenesis, differentiation, and ECM remodeling [229-233]. DDRs display an affinity for newly synthesized, proteolytically degraded, or damaged collagens over intact fibrils due to the availability of DDR-binding sites [227, 234]. The ramification of DDR partiality towards collagen-binding may be its contribution to up-regulated expression and activity of matrix metalloproteinases (MMPs) [235, 236].

The MMPs are tightly regulated through transcriptions or proteolytic cleavages and are degrading proteases for ECM components [237]. And the DDR exertion over MMPs causes tissue remodeling, facilitation of cell migration, organ development encroachment, and diseases [238, 239]. Thus, numerous human diseases, such as inflammatory disorders, fibrosis, cancer, and neurodegeneration, can be linked back to DDRs [238]. For example, many studies are now underway in deciphering DDRs’ role in cancer. Collagens—a major ECM component—are shown to undergo extensive remodeling during tumor progression. And over-expression of DDR1 and DDR2 in multiple forms of cancers suggests that they are involved in the proliferation and migration of tumor cells [240]. But our very limited understanding of DDR activation *in vivo* begs for further studies before we can fully invest in DDR inhibitory strategies for cancer treatment [241]. In neuro-degenerative disorders-Alzheimer's and Parkinson's -DDR up-regulation was observed in post-mortem brains. Cell loss prevention was achieved via the lentiviral delivery of short hairpin RNA knockdown. These knockdowns exhibited reduced levels of α-synuclein, tau, and β-amyloid [242] and also significantly altered brain immunity by reducing the level of triggering receptors expressed on myeloid cells (TREM)-2 and microglia [243]. Such results are generating much excitement over the DDR as the new favorite target in tackling neurodegenerative disorders [244].

DDRs have a crucial role in aging. Many diseases we associate with aging were shown to have DDR involvement in their disease mechanisms. Yet, little is known as to how. It is, therefore, crucial to study the basic mechanisms of DDRs. Concurrently, targeted DDR deletions in mouse models are extending our understanding of the role of DDRs in disease progressions. We found that in many instances, the DDRs have a positive contribution to the pathologies. Anti-DDR therapies—such as targeted RNA interference (RNAi)-based delivery—are therefore being developed for diseases with limited treatment options [241].

#### 2.4.2. The Integrin receptor family

The communication between cells and the ECM predominately passes through the integrin [76]. As the name implies, this family of proteins integrates intra- and extracellular cues. In the majority of cell types, integrins connect the extracellular space to the cytoskeleton and establish a mechanotransduction axis. And unlike DDRs, integrins display a variable affinity for their ligands depending on their activation state. Integrin receptors are present as α-β heterodimers (Fig. 1B). The subunits dimerize in a calcium-dependent manner in the endoplasmic reticulum (ER) and connect to the plasma membrane (PM) in their inactive conformation [136]. Multiple subunit combinations and small recognition motifs license integrins to bind to macromolecular ligands like collagens, laminin, fibronectin, thrombospondin, osteopontin, vitronectin, fibrillin, fibrinogen, and cell surface receptors [69].

The integrin relays the signal bidirectionally. It can transmit information inside-out through the binding of adapter proteins to the cytoplasmic domain of the β subunit—thereby favoring the active integrin conformation [106, 122, 133]. Integrin receptors can also signal outside-in by clustering together. Integrin receptors bind to common extracellular ligands [67] or through external mechanical forces causing integrins to alter their conformations [27, 28]. One example of the crucial role of the integrin is fibroblasts migrating along collagen fibers to respond to wounded tissue [88] (Fig. 1A). The integrin activation modulates actin filament dynamics by controlling the activity of Rho GTPases [32, 37]. The Rho GTPases-especially the Rho, Rac, and the Cdc42—regulate the interaction between the actin cytoskeleton and ECM adhesion [32] by selectively acting through multiple downstream effectors, including Rho-associated coiled-coil-containing protein kinase (ROCK) and PAK [238] (Fig. 1B).

Most known age-dependent diseases share a commonality of cellular stress response called senescence. Previous understanding defined senescence as an endpoint to stress, causing cells to lose their ability to proliferate [245]. And in *C. elegans* and *D. melanogaster*, senescence has been associated with the integrin-signaling complex components. While it was demonstrated that complete deletion of integrin-linked kinase (ILK) caused lethality, moderate ILK reduction seems to have a positive function in longevity [246, 247]. Notably, *C.elegans* also had an increased lifespan without much loss in cytoskeletal integrity [246], and RNAi screening has revealed that *pat-4*/ILK alongside *pat-6*/Parvin depletion caused lifespan extension [246, 248]. Furthermore, the binding partner of the ILK-a heterozygous mutation in β1-integrin-delayed aging and mortality in *Drosophila* [247]. In mice, cytoskeletal remodeler Coronin-7 (*Coro7*) is associated with a longer lifespan [249]. In human fibroblasts-via matricellular cell adhesive protein CCN1-β1 integrin caused senescence in wound healing [250]. Furthermore, β3 integrin induced senescence through activation of the transforming growth factor (TGF)-β pathway while its knockdown overcame senescence [251]. All these findings point to novel therapeutics using down-regulation of targeted integrin signaling to induce a delay in cellular senescence [252].

### 2.5. Matrisome homeostasis and turnover

#### 2.5.1. Definition of the matrisome

The biochemistry of the insoluble ECMs eluded scientists for years. However, with the emergence of proteomic tools, Martin et al. shed light upon the composition of the ECM in 1984-coining the term “matrisome” for supramolecular complex components of the matrix responsible for the formation of functional units of the ECM [253].

Since its initial days, bioinformatics tools such as the matrisome project and the MatrisomeDB-in combination with novel experimental approaches of combining gene orthology, protein structure analysis, and exhaustive curation of the literature-have extended the definition of the matrisome to include 1027 human genes encoding the ECM and ECM-associated proteins that induce remodeling through its interactions [167, 254, 255].

From data compiled *in silico* and *in vivo,* matrisome was divided into two subcategories. The core matrisome proteins represent structural elements of the ECM and comprise ECM glycoproteins, collagens, and proteoglycans. The matrisome-associated proteins encompass ECM-affiliated proteins, ECM regulators, and secreted factors [167, 254].

The matrisome has been defined for 1027 genes in humans [255], 1110 genes in mice [255], 1002 genes in zebrafishes [256], 706 genes in Japanese quails [257], 641 genes in *Drosophila* [258], 719 genes in *C. elegans* [259], and 256 genes in planarians [260]. Although the matrisome accounts for approximately 4 percent of their total genome, mutations or variants in these matrisome genes result in about 3-10% of the overall phenome (*i.e.*, a spectrum of all phenotypes), which consists of 11666 phenotypes of the above-listed species [261]. These findings show that matrisome genes are highly conserved throughout species-making model organisms such as the *C. elegans* ripe for studying biological mechanisms, pathology, and drug development.

#### 2.5.2. Matrisome in health and disease

The collective consensus on the definition of matrisome by the scientific community allowed the identification of previously unsuspected ECM proteins to be discovered, and bioinformatics predictions were able to forecast strongly adverse outcomes of diseases such as cancer [255, 262-266].

Yuzhalin and colleagues analyzed that overexpression of matrisome was observed-with nine commonly upregulated genes-in many patients suffering from a diverse array of cancers. Furthermore, overexpression of these gene signatures was linked to epithelial-mesenchymal transition, angiogenesis, hypoxia, and inflammation in patients [267].

Defects in the matrisome have been linked to a vast array of pathologies because the matrisome not only encodes many structural components of the ECM but also because its molecules act as a control to cell adhesion, migration, proliferation, differentiation, and senescence. Any defects inherited or acquired in the matrisome are susceptible to causing havoc at the cellular and tissue level and may lead to various disease development and progression [268]. As such, matrisomal properties can then be exploited as novel biomarkers for these diseases [254, 255]. And the matrisome can also be employed as an effective therapeutic targeting tool by affecting the ECM through post-translational modifications, synthesis, remodeling, and intervening in the receptors [255, 268].

In the future, matrisome/ECM engineering may also solve the shortage of organs for the patients waiting on the transplant list. Various ECM receptors are stem cell markers such as laminins. The laminin is thought to affect stem cell differentiation and pluripotency. Such ECM property can perhaps be used to reconstruct or build whole functional organs in the future [255, 269-273].

#### 2.5.3. Matrisome during aging and longevity

The turnover of the ECM requires strict regulation and coordination. Even though the ECM appears static on the macroscopic level, it constantly undergoes remodeling and maintains a precarious balance between collagen synthesis and degradation [274]. Osteoarthritis may be observed if the balance favors degradation [275]. And excessive synthesis or insufficient degradation resulting in high matrix density has been attributed to fibrotic and tumor tissues [276]. Furthermore, aging itself can drive the extensive remodeling of the muscular ECM, thereby impairing muscle stem cell function and contributing to the loss of muscle regeneration during older age [277].

The matrisome incorporates all identified proteins present in the extracellular region that is not part of the cell membrane [167] and consists of many stable proteins. Processes like growth to accommodate the altered size of the tissue, vascularization, and wound repair demand a fast turnover.

The continuous turnover is evidenced by the continuing expression of matrisome components well after the matrix is formed [274] despite the involved proteins displaying very long half-lives. The qualitative and quantitative composition of the ECM translates to the ideal mechanostability and permeability, which are in direct connections to multiple pathologies like tissue fibrosis, kidney function, cancer metastasis, and others.

**Figure 2.**
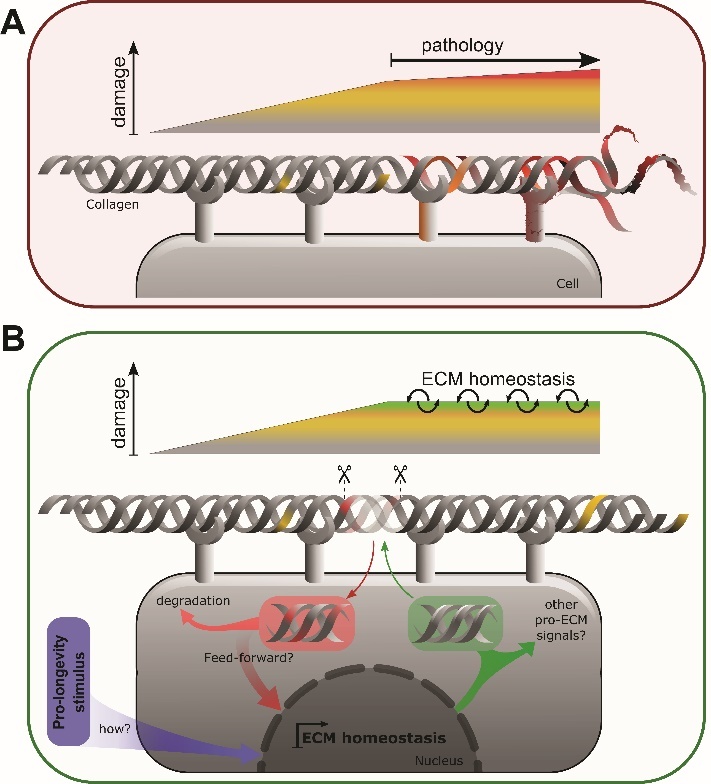
Proposed matrisome aging process through the loss of homeostasis. (A) A schematic representation of the matrix deterioration and inflammation observed in aged ECM. (B) Hypothetical ECM homeostasis process activated in longevity. The endo- and exocytosis steps of ECM components produced and taken up by the surrounding cells are also depicted. Currently, neither the stimuli activating homeostatic ECM turnover (violet) nor the genes regulating controlled ECM remodeling (green, red) in this proposed longevity assurance pathway have been identified.

In addition, collagens and other structural elements are likely recycled to prevent age-induced modifications and maintain a youthful ECM (Fig. 2). If not replenished, old matrices lose their ability to respond to mechanical stresses and become less elastic due to post-translational modifications (Fig. 2). The matrisome forms massive, insoluble, and heavily modified protein structures, and maintaining their function thus is a challenge.

#### 2.5.4. Matreotype

As stated above, the matrisome encompasses a broad spectrum of genes that encode all the ECM and ECM-associated proteins. However, not all matrisome genes are expressed at the same time in the same tissue. For instance, in single-cell RNA-sequencing analysis of chicken and mouse limb development, the core matrisome comprises 2% of the entire cellular transcriptome of a given cell but suffices to predict cell type [111]. This strengthens the statement that “each cell type makes its own ECM” [276]. Analysis of the human Genotype-Tissue Expression (GTEx) data found ECM-related gene expression on different physiological statuses, confirming previous findings of increased matrix protease and decreased collagen expression with age [278]. But because each tissue type showed differences in ECM gene expressions, and temporal association differed depending on tissue types, there was an imperative need for introducing a new conceptional framework to accurately reflect organismal phenotype and physiological states in matrisomal gene expression. Furthermore, as discussed above, healthy or diseased tissue can be identified by proteomics of the surrounding ECM, for instance, for cancer [129, 263, 279]. Thus, we introduced the concept of the matreotype [129]. The matreotype is the snapshot of the ECM composition associated with or caused by a phenotype or physiological state [129].

Aging is the state of the declining rate of remodeling of the ECM and slowing of matrisome component synthesis to retain homeostasis (Fig. 2A). This decline is demonstrated by the differences seen in the matreotypes of young and old ECMs. Longevity interventions prolong collagen homeostasis, which is required and sufficient for extending lifespan [82], suggesting the hypothesis that longevity interventions prolong ECM homeostasis to promote healthy aging (Fig. 2B).

Thus, ultimately, understanding and identifying different stages of matreotypes can be instrumented as key markers to confirm the prognosis of diseases and the development of personalized medicine [129].

**Figure 3.**
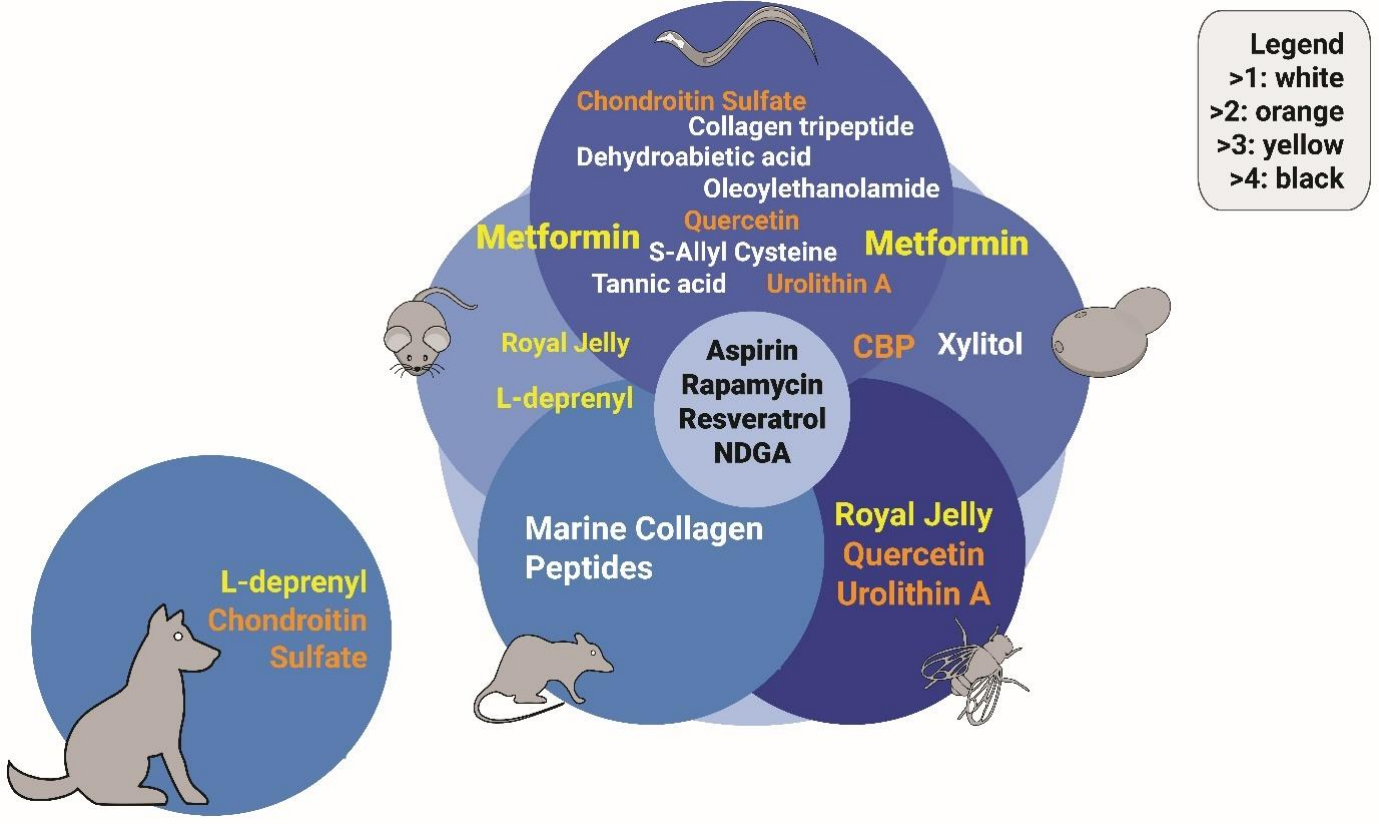
Compounds promoting ECM homeostasis and longevity. Representation of compounds that affect extracellular matrix proteins and have been shown to increase healthspan and/or lifespan in at least one species. Colors indicate the number of species a given compound has shown to promote longevity. The source data is from Aging Cell. 2021 Sep;20 (9): e13441. doi: 10.1111/acel.13441. PMID: 34346557.

### 2.6. Longevity interventions promoting ECM homeostasis

The matreotype has the potential to be utilized for interventions promoting health during aging. One observation is that chronic age-related pathologies heavily remodel the ECM during disease development and progression [280]. Not so surprising is that interventions that slow aging and prevent chronic diseases also rebalance pathological ECM remodeling [280]. Interestingly, analyzing chromatin immunoprecipitation (ChIP) sequencing and transcriptomic data, we found that the 12 most established longevity-promoting transcription factors (*i.e.*, CREB1, FOXO1,3, GATA1,2,3,4, HIF1A, JUN, KLF4, MYC, NFE2L2/Nrf2, RELA/NF-κB, REST, STAT3,5A, and TP53/p53), directly and indirectly transcriptionally regulate ECM genes [280]. Thus, changing the matreotype may promote ECM homeostasis.

Furthermore, the observation that pro-longevity compounds in model organisms also alter the matreotype may hold the key to attaining concrete biomarkers in the future (Fig. 3) [278]. Armed with this observation as our launchpad, we used age-stratification of human transcriptomes to define matreotypes related to aging-with youthful matreotype as a benchmark to screen for geroprotective drug candidates *in silico*. Drug candidates were then screened on *C. elegans* using prolonged collagen expression as a surrogate marker *in-vivo* and eliminated false positive drug candidates. Using this novel approach, we were able to improve drug uptake and reconcile contradictory reports of existing drugs. We also found and validated new compounds that rejuvenated collagen homeostasis and increased lifespan. Thus, not only does the matreotype have the potential to quantify human aging, but through the employment of extracellular matrix homeostasis, we may facilitate the discovery of novel pharmacological interventions to promote healthy aging [129, 278].

### 2.7. ECM dynamics as a hallmark of aging?

As Hanahan and Weinberg defined the hallmarks of cancer [281], López-Otín and colleagues have defined the hallmarks of aging [10]. However, Gems and de Magalhães, in their critique of the hallmarks of aging, argued that López-Otín et al. fail conceptually to provide a concrete explanatory paradigm [282]. Fedintsev and Moskalev, as well as Gorisse and colleagues, proposed stochastic non-enzymatic modification on collagens and other ECM proteins as a missing hallmark of aging [283, 284]. Furthermore, the 2022 proposal of the new hallmarks of aging worked out by the scientific community at the Copenhagen aging meeting suggested including “altered mechanical properties” [285]. Thus, this begs the question of whether to include ECM dynamics and mechanotransduction as one of the hallmarks. Although, undoubtedly, two of the three criteria of a hallmark are met (*i.e.*, (i) it should manifest during normal aging and (ii) experimental aggravation must accelerate aging), the last one requires more experimental evidence. The third criterion states that its experimental amelioration should retard the normal aging process and, thus, increase a healthy lifespan [10]. Although there is tantalizing evidence in *C. elegans* for this third criterion of collagen homeostasis may be ameliorating collagen stiffness [286] and being required and sufficient for longevity [82], the experimental evidence in mammals is missing. And per definition, to qualify as one of the hallmarks, mammalian aging evidence is essential. This last criterion might be the most difficult to provide evidence for. Thus far, altering collagen turnover, either by altering metalloprotease activity or changing cleavage sites in collagens, counterintuitively resulted in premature and accelerated aging instead of slowing aging [222, 223]. Indirect but tantalizing evidence for at least mouse brain rejuvenation was demonstrated by administrating human umbilical cord plasma, which is enriched in tissue inhibitor of metalloproteinase TIMP2 that inhibits MMPs and alters ECM remodeling Castellano et al. This might suggest a more transient and intermediate alteration of ECM remodeling might be a strategy to increase lifespan. Thus, demonstrating that any intervention that targets the ECM is sufficient to increase mammalian aging is the last piece of evidence missing for classifying the dysfunctional ECMs as a hallmark of aging. As has been demonstrated by how the loss of homeostasis impacts various pathologies in the previous sections, it looks like ECM homeostasis has the potential to become a hallmark of aging with further experimental evidence. It is clear that the extracellular matrix dynamics influence all hallmarks of cancer [287] and aging [283, 284].

## 3. Concluding remarks

The ECM-associated pathologies encapsulate the damage potential of deregulated ECM turnover. Both the process of ECM production and its degradation have the potential to result in catastrophic tissue failure.

In the case of the skin, it appears to be an age-dependent reduction in synthesis, and in the remaining organs, an age-dependent reduction in targeted degradation causes pathologies. Improved homeostasis through the bidirectional nucleus to the PM and the ECM signaling could alleviate this issue. Potentially, accelerating collagen turnover rate during middle age may delay the point of catastrophic damage accumulation and the ECM homeostasis breakdown in older individuals.

We currently lack a comprehensive understanding of the role of the ECM across multiple phenotypes, such as aging and the association of the matrisome with chronic diseases—especially the regulation of matrisome genes in aging and longevity.

Conceivably, the underlying root cause of many age-associated pathologies could be the progressive loss of ECM homeostasis that comes with age. Preliminary evidence from longevity studies in model organisms has implicated such a mechanism [82]. However, further studies are necessary to give conclusive transferable answers.
